# Supplementary material for: Hepatic Sdf2l1 controls feeding-induced ER stress and regulates metabolism
Source: Nat Commun. 2019 Feb 27;10:947. doi: 10.1038/s41467-019-08591-6 (PMC6393527; doi:10.1038/s41467-019-08591-6)
Supplement: Supplementary file 1 — Supplementary Information [file 41467_2019_8591_MOESM1_ESM.pdf]

Supplementary Figure Legends

Supplementary Fig. 1

ER stress responses and protein synthesis during feeding.

(a) Expression of genes associated with ER stress, analyzed by RT-PCR. *uXbp1*: unspliced *Xbp1*, encoding XBP-1u.

(b-d) Molecules involved in ER stress responses and protein synthesis markers, analyzed by Western blotting, using (b, c) total lysates and (d) immunoprecipitates, in the liver of wild-type mice in an *ad libitum*-fed state or 0 to 6-hour refed states after 24 hours of fasting.

(e, f) RT-PCR analysis of *Sdf2l1* expression in insulin-targeted organs of wild-type mice (n = 4), (e) in a 24-hour fasted state, normalized by the expression level in the liver, and (f) in a 6-hour refed state, normalized by the expression level in a fasted state.

Values of the data are expressed as mean ± SEM. *P < 0.05, **P < 0.01, ***P < 0.001. Unpaired 2-tailed t-test was used for assessment.

Supplementary Fig. 2

Contribution of protein intake and insulin signaling to feeding-induced ER stress responses.

ER stress markers analyzed by (a, c, e) RT-PCR and (b, d, f) protein synthesis markers analyzed by Western blotting, in mice (a, b) fed with protein- or lipid-free chow, (c, d) treated with STZ in advance, and (e, f) treated with STZ in advance and fed with protein-free chow (n = 3-5 for each). In (b, f), the lanes were run on the same gel but were noncontiguous.

Values of the data are expressed as mean ± SEM. *P < 0.05, **P < 0.01. (a) One-way ANOVA was used, and (b-f) unpaired 2-tailed *t*-test was used for assessment.

Supplementary Fig. 3

Expression regulation and functions of Sdf2l1.

(a) RT-PCR analysis of *Sdf2l1* and *Tmed10* expression in Fao cells after treatment with tunicamycin or thapsigargin for 16 hours (*n* = 3-4).

(b) *Sdf2l1* promoter assay in Fao cells, with tunicamycin treatment using Ad-Sdf2l1-Luc (*n* = 3).

(c) *In vivo* imaging, after intravenous administration of Ad-Sdf2l1-Luc in wild-type mice (3.0 x 10^6 PFU/g BW), comparing the *Sdf2l1* promoter activity in the liver, in a fasted state and a 4-hour refed state (*n* = 3).

(d) *Sdf2l1* promoter assay in Fao cells, with thapsigargin treatment using reporter plasmids (*n* = 3).

(e) Alignment of the *Sdf2l1* promoters including the region of interest among species, using ClustalW2 (http://www.ebi.ac.uk/Tools/msa/clustalw2/).

(f) Alignment of the region of interest in the *Sdf2l1* promoter and ERSEs, having ATTGG (underlined) in common.

(g) RT-PCR in Fao cells, with *Xbp1* or *Atf6* knocked down (*n* = 3).

(h) ChIP assay for analysis of binding with the *Hspa5* promoter, in a 24-hour fasted state and a 3-hour refed state (*n* = 3). The relative expression levels were normalized by the upstream region (-4.5kb) and then by control IgG.

(i) Schematic description of Pmt2-Sdf2l1-HA fusion protein and Sdf2l1-FLAG protein. This figure was created by T. S.

(j) A yeast strain of KNY51 (*der1Δ, pmt2Δ, GAL1-Δpro*), expressing mutant GAL1-Δpro, which could not be degraded by ER stress-associated degradation (ERAD) due to lack of *DER1*, nor be *O*-mannosylated in a compensatory manner due to lack of *PMT2*, was transfected with Pmt2-HA or Pmt2-Sdf2l1-HA. Expression of restored Pmt2 and *O*-mannosylated Δpro was analyzed by Western blotting. The lanes were run on the same gel but were noncontiguous.

Values of the data are expressed as mean ± SEM. *P < 0.05, **P < 0.01. (a, d) One-way ANOVA was used, and (b, c, g, h) unpaired 2-tailed *t*-test was used for assessment.

Supplementary Fig. 4

Generation of *Sdf2l1*-floxed mice.

(a) Construct of *Sdf2l1*-floxed mice, including scheme of Southern blotting.

(b) Scheme of genotyping PCR.

(c) Results of Southern blotting and genotyping PCR of obtained mice, after crossing with *ACTB-FLPe* for elimination of *Neo*.

(d) Western blotting of *Sdf2l1*-floxed MEF cells infected with adenoviruses.

Supplementary Fig. 5

Knocking down of *Sdf2l1* and related molecules *in vitro*.

(a, b) Knocking down in primary hepatocytes, analyzed by (a) RT-PCR (n = 4) and (b) Western blotting of microsomal fractions.

(c) Primary hepatocytes were infected with Ad-Ins^{C96Y}, with *Sdf2l1* or *Syvn1* knocked down, whose lysates were immunoprecipitated for Western blotting. Multi: multiubiquitinated insulin, Mono: monoubiquitinated insulin.

(d) RT-PCR to analyze ER stress marker gene expression in NIH/3T3 cells with *Sdf2l1* and/or *Syvn1* knocked down, treated with tunicamycin treatment (n = 3).

Values of the data are expressed as mean ± SEM. *P < 0.05, **P < 0.01. Unpaired 2-tailed *t*-test was used for assessment in (a), whereas one-way ANOVA was used for assessment in (d).

Supplementary Fig. 6

Phenotypes of the knockdown model of *Sdf2l1*.

(a-d) Knocking down of *Sdf2l1* in wild type mice: (a) Body weight; (b) expression of genes associated with ER stress, analyzed by RT-PCR; (c, d) Western blotting to analyze insulin signaling in (c) the liver with immunoprecipitates and (d) skeletal muscle with total lysates; (e) fasting plasma free fatty acid levels (n = 10). *Mbtps1* encodes S1P, and *Acacb* encodes ACC2.

Values of the data are expressed as mean ± SEM. *P < 0.05, **P < 0.01. Unpaired 2-tailed *t*-test was used for assessment.

Supplementary Fig. 7

ER stress responses and protein synthesis in obesity and diabetes models.

(a, b) Analysis of molecules involved in ER stress responses and protein synthesis markers by Western blotting, using (a) immunoprecipitates, and (b) total lysates, in the liver of *db/db* mice in 0 to 6-hour refed states after 24 hours of fasting.

(c) ChIP assay for analysis of binding with the *Hspa5* promoter, in a 1-hour refed state after 24 hours of fasting (*n* = 3). The relative expression levels were normalized by the upstream region (-4.5kb) and then by control IgG.

(d, e) ER stress markers analyzed by RT-PCR in (d) *ob/ob* mice, and (e) mice fed with high-fat diet for 8 weeks (*n* = 4 for each).

Values of the data are expressed as mean ± SEM. *P < 0.05, **P < 0.01. Unpaired 2-tailed *t*-test was used for assessment. NC: normal chow, HFD: high-fat diet.

Supplementary Fig. 8

Phenotypes of *db/db* mice with chaperones restored.

(a-c) *db/db* mice were administered with Ad-FLAG-XBP-1s intravenously (2.0 x 10^7 PFU/g BW), for enhancing the gene expression in the liver (*n* = 3-5): (a) gene expression by RT-PCR, (b) *ad libitum*-fed plasma glucose, and (c) the AUC of plasma glucose levels in ITT, after intraperitoneal injection of human regular insulin (3.0 U/kg BW).

(d-j) Restoration of Sdf2l1, (d, e) alone, and (f-j) in combination with BiP: (d, h) body weight, (e, f) RT-PCR, (g) Western blotting, (i) products of plasma glucose and insulin levels in an *ad libitum*-fed state, and (j) plasma glucose levels in ITT.

Values of the data are expressed as mean ± SEM. *P < 0.05, **P < 0.01. (d, e) Unpaired 2-tailed *t*-test was used, and (f, h-j) one-way ANOVA was used for assessment.

Supplementary Fig. 9

ER stress responses in human subjects.

Comparison of ER stress marker gene expression between diabetic (*n* = 25) and matched nondiabetic subjects (*n* = 25), analyzed by RT-PCR. Data are shown in box and whisker plots.

Supplementary Fig. 10

Schematic description of our hypothesis on physiological and pathophysiological roles of Sdf2l1-centered ER stress responses in the liver. This figure was created by T. S.

Supplementary Fig. 11

Uncropped images of the blots included in the figures.

Supplementary Figure 1

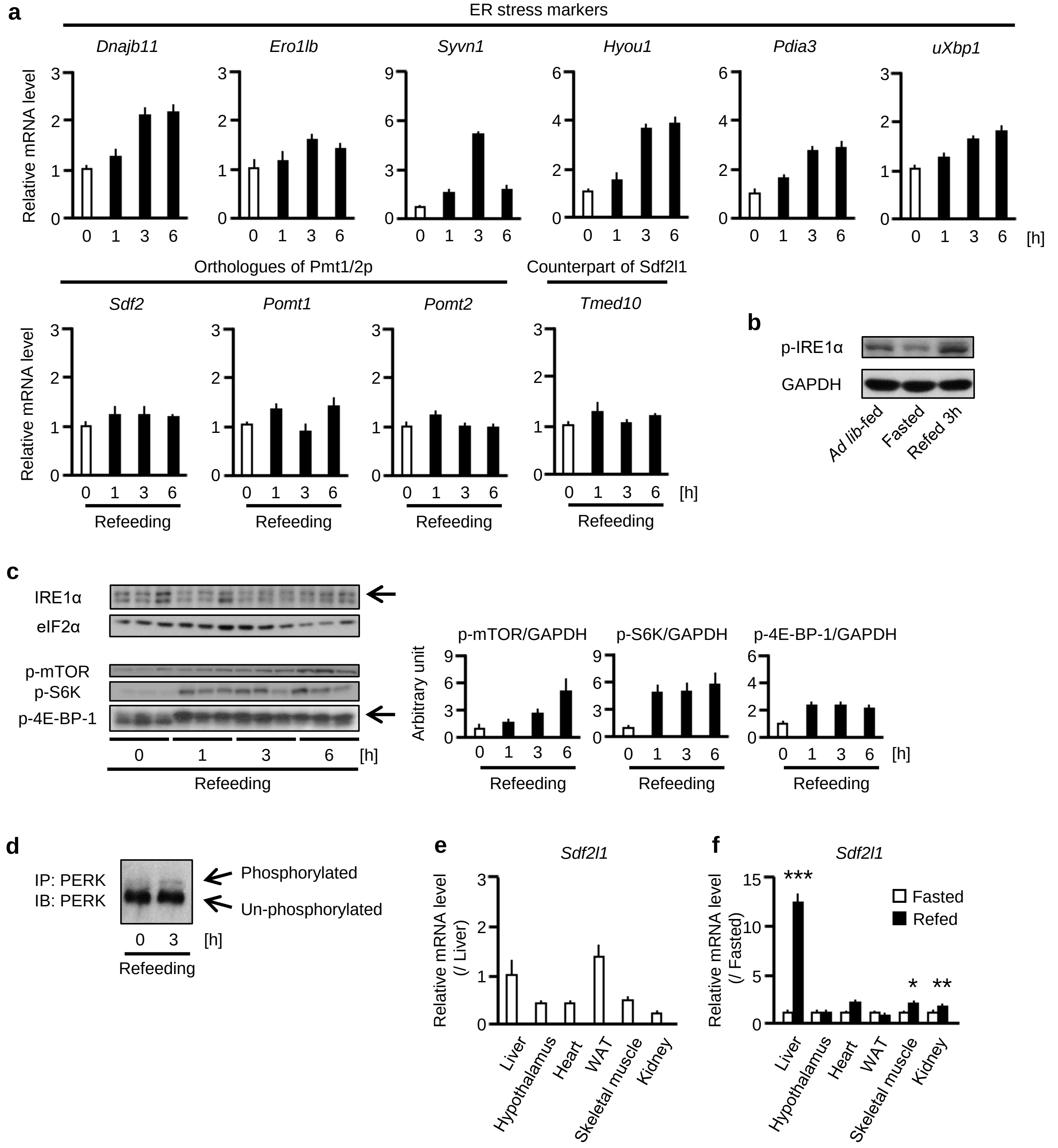

Supplementary Figure 2

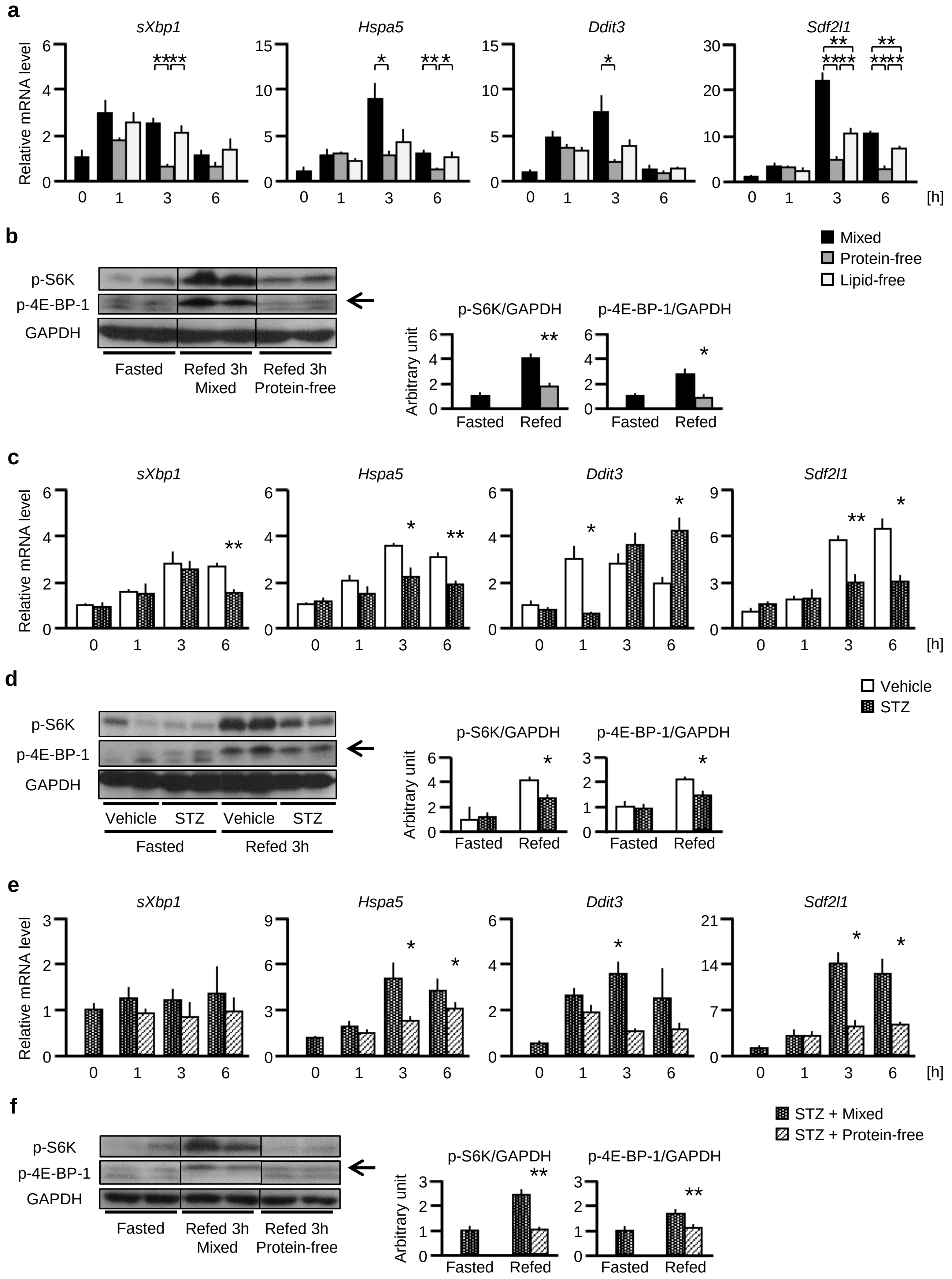

Supplementary Figure 3

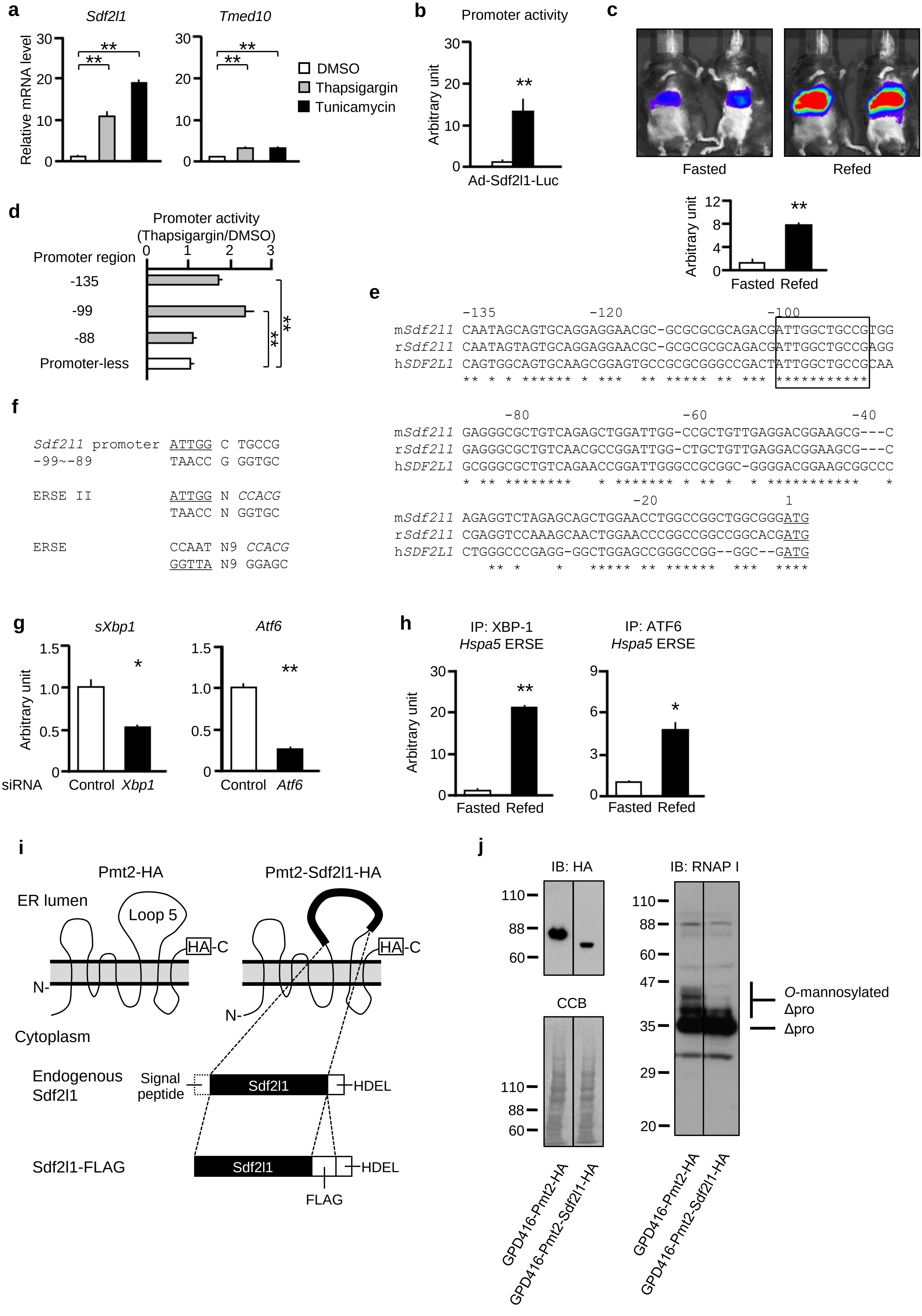

Supplementary Figure 4

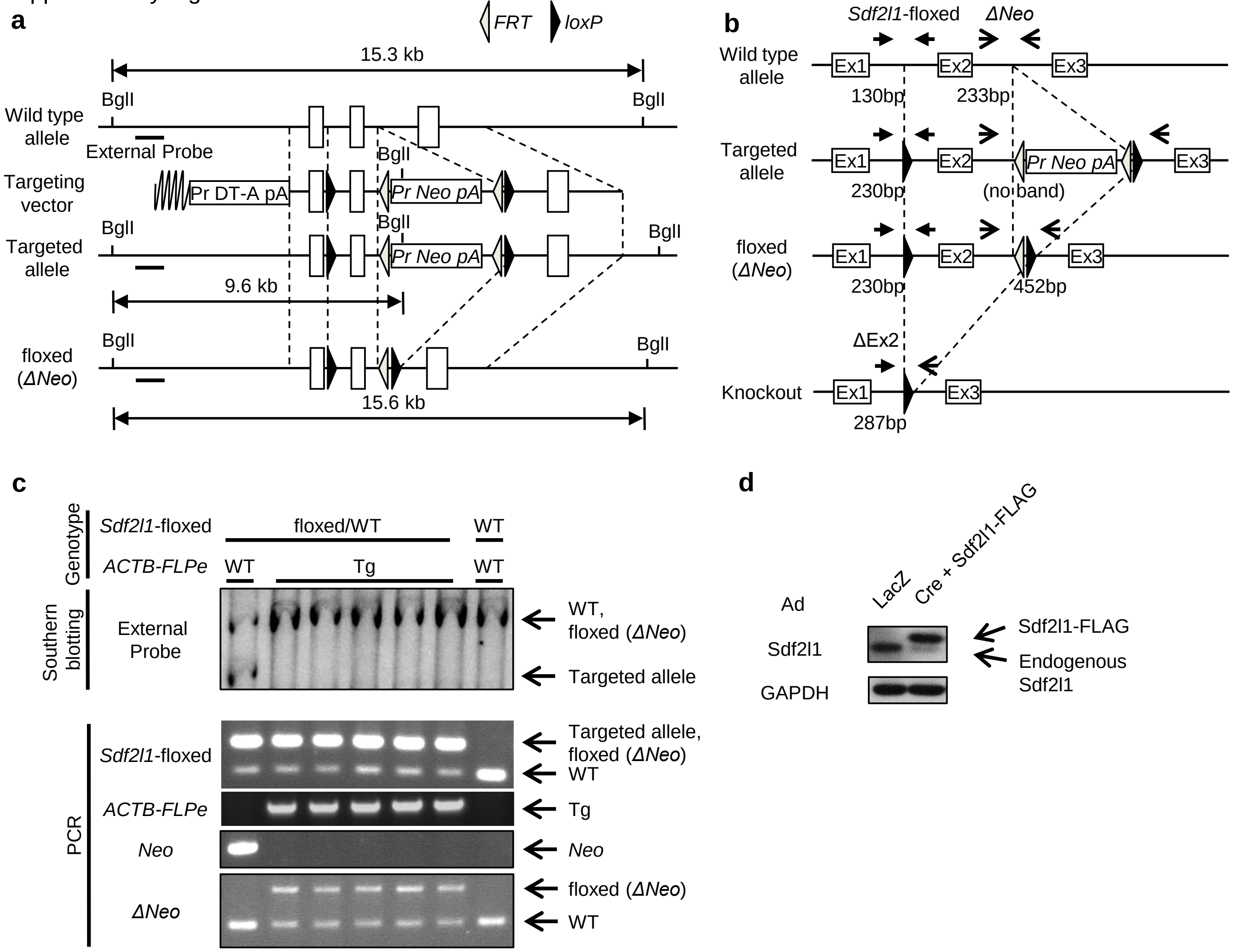

Supplementary Figure 5

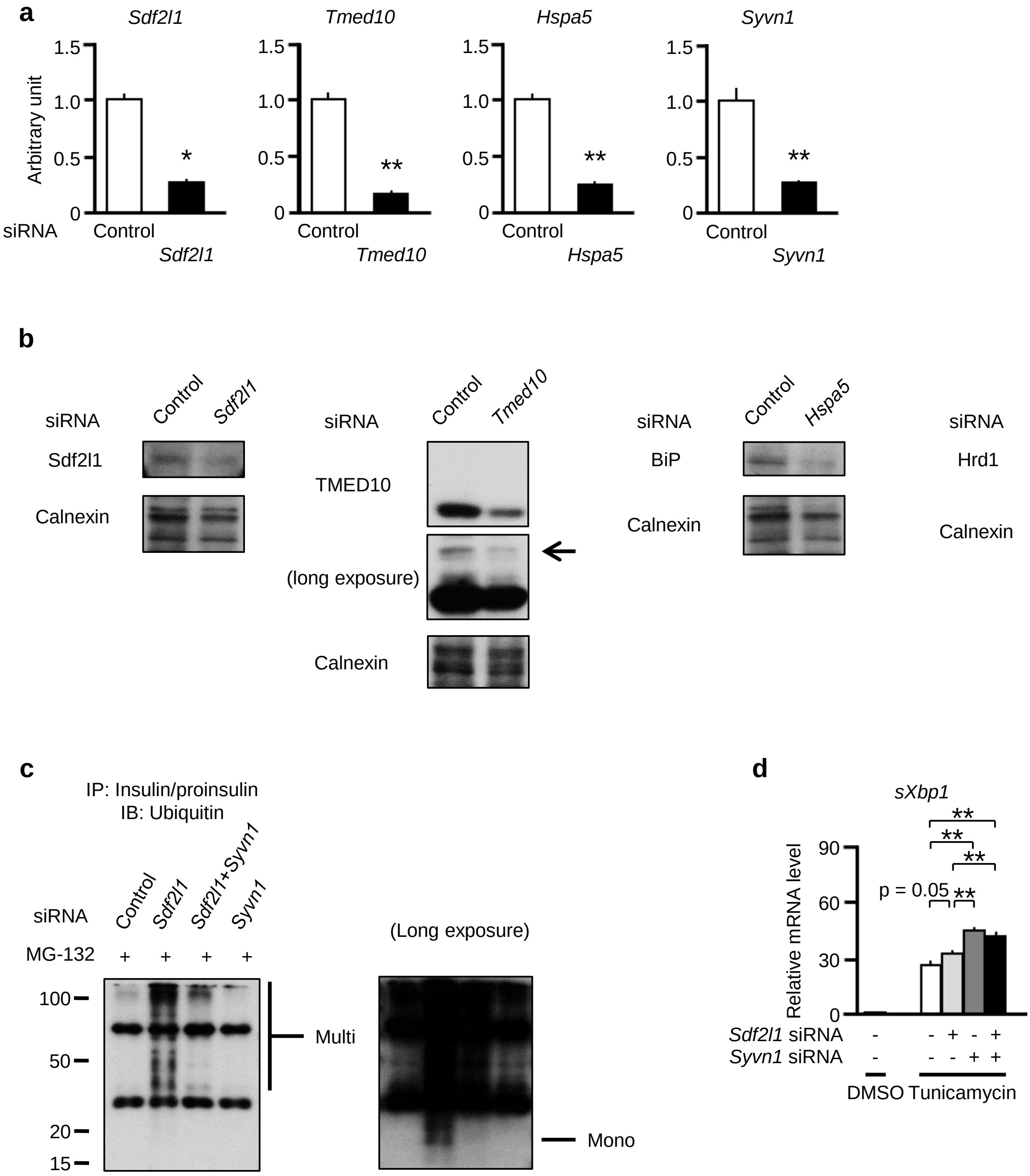

Supplementary Figure 6

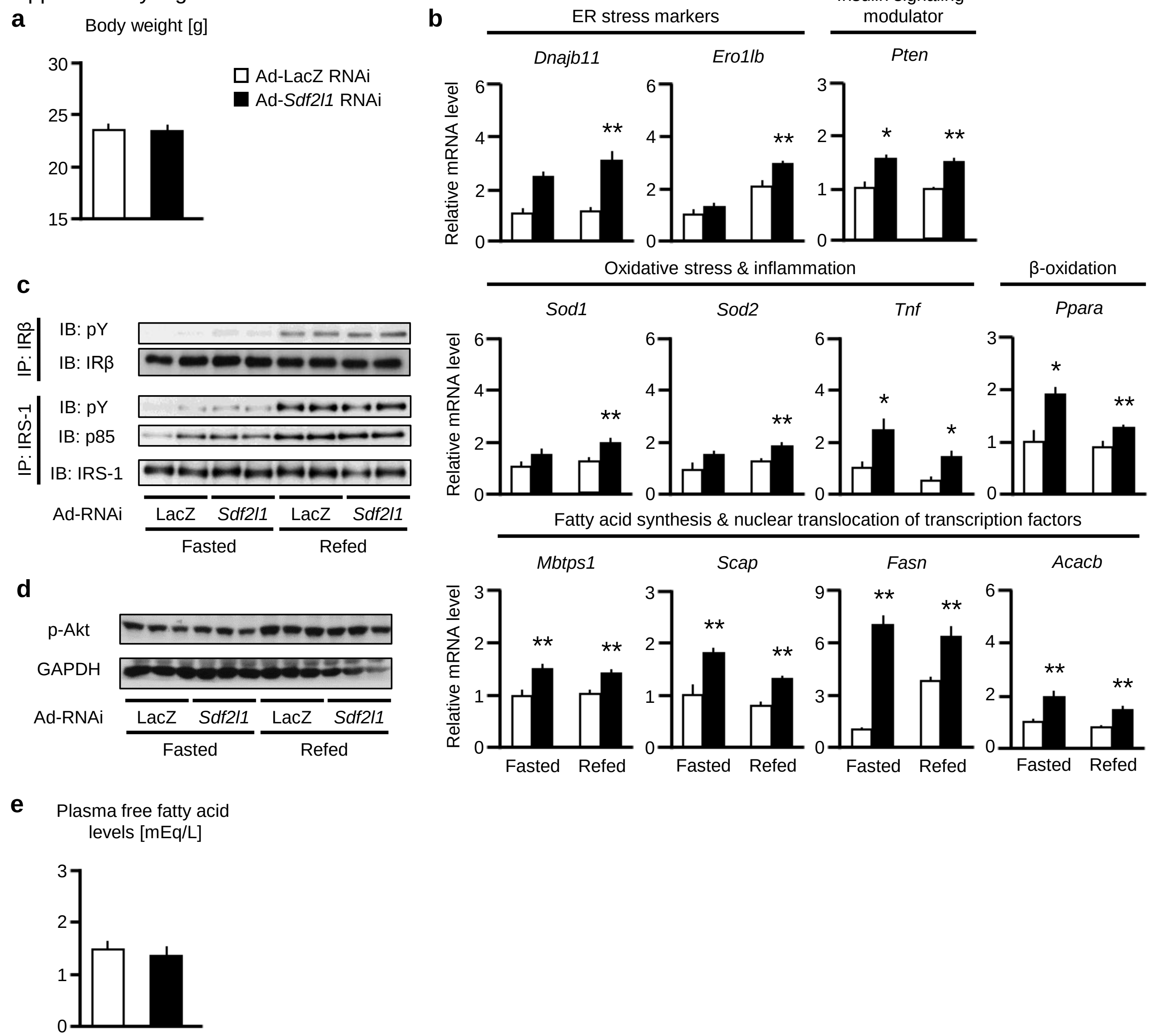

Supplementary Figure 7

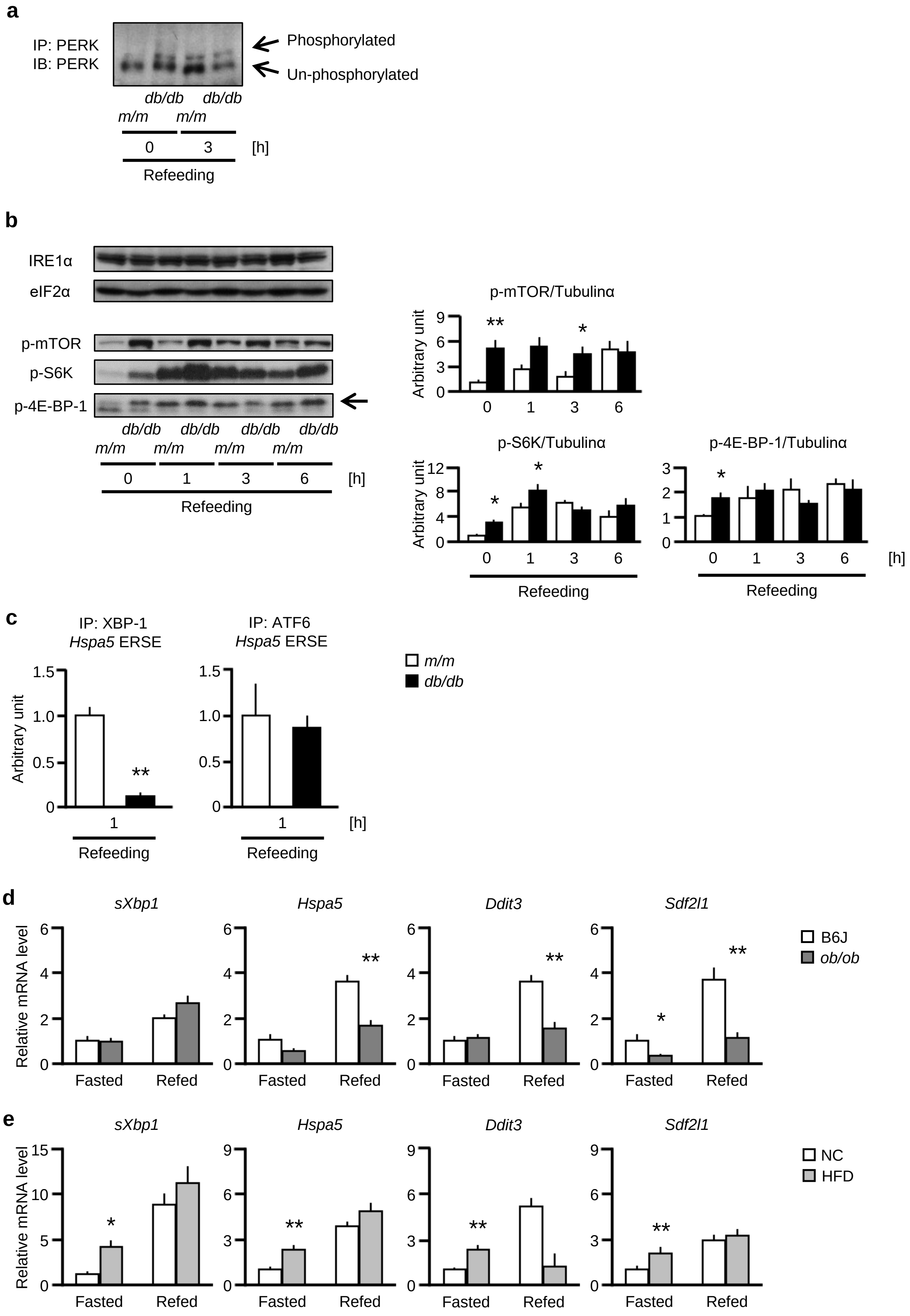

Supplementary Figure 8

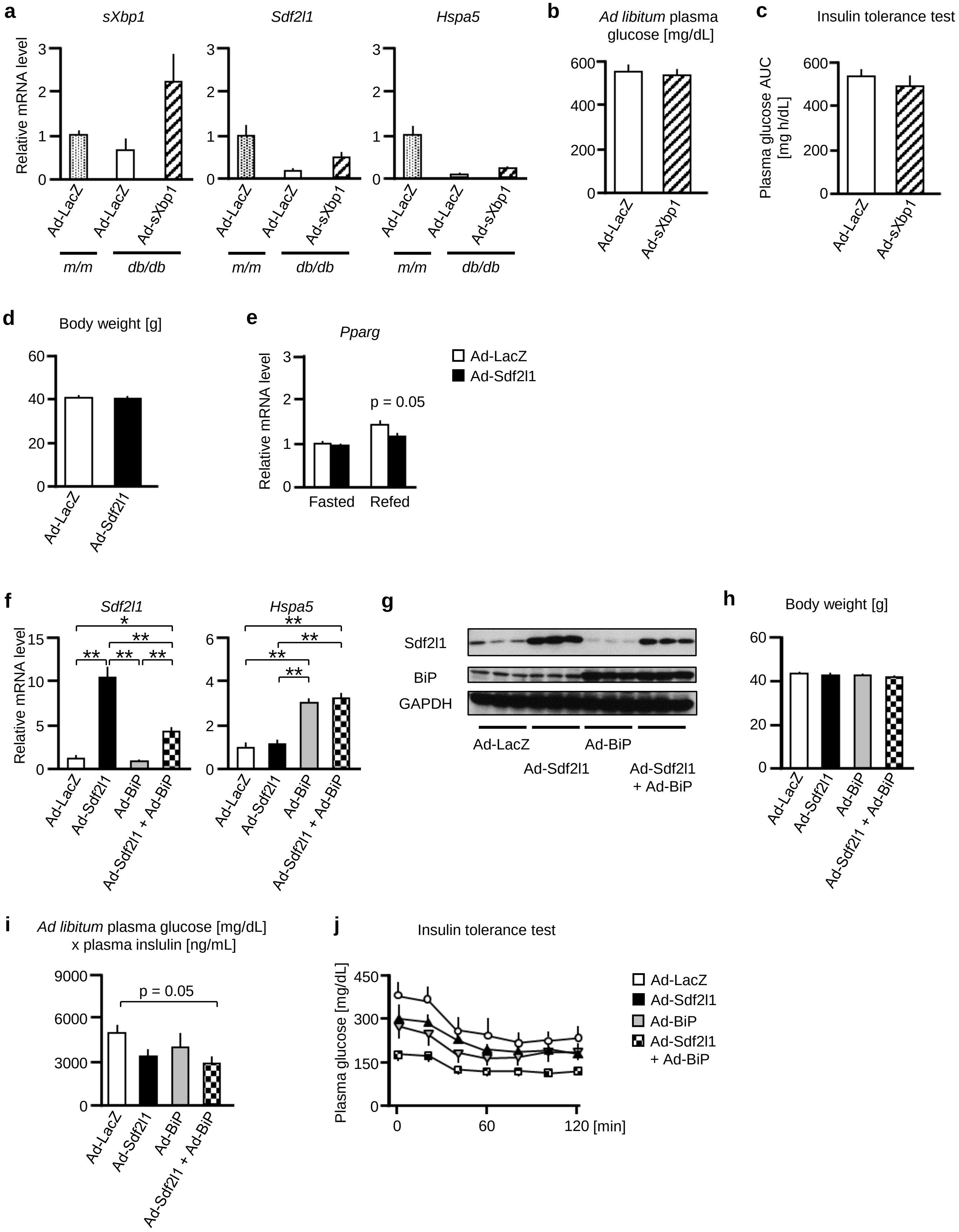

Supplementary Figure 9

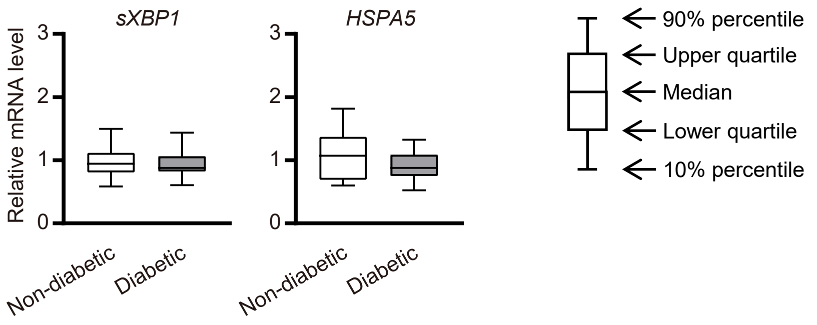

Supplementary Figure 10

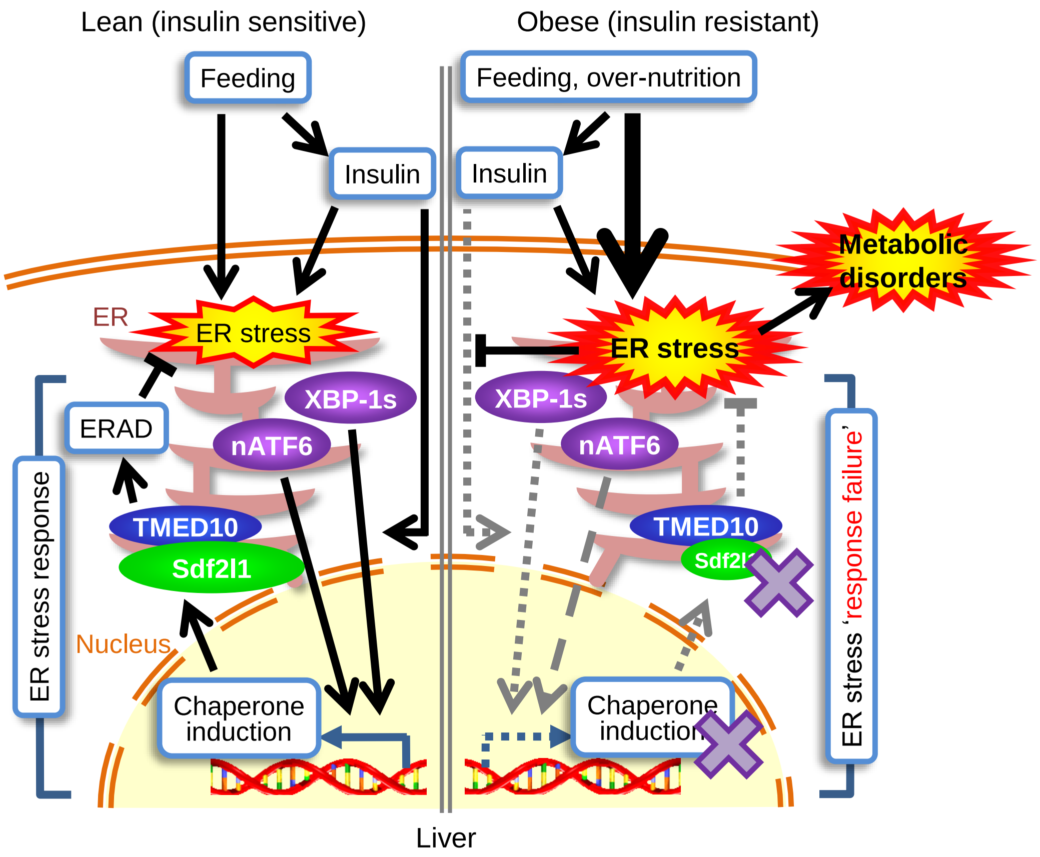

Supplementary Figure 11

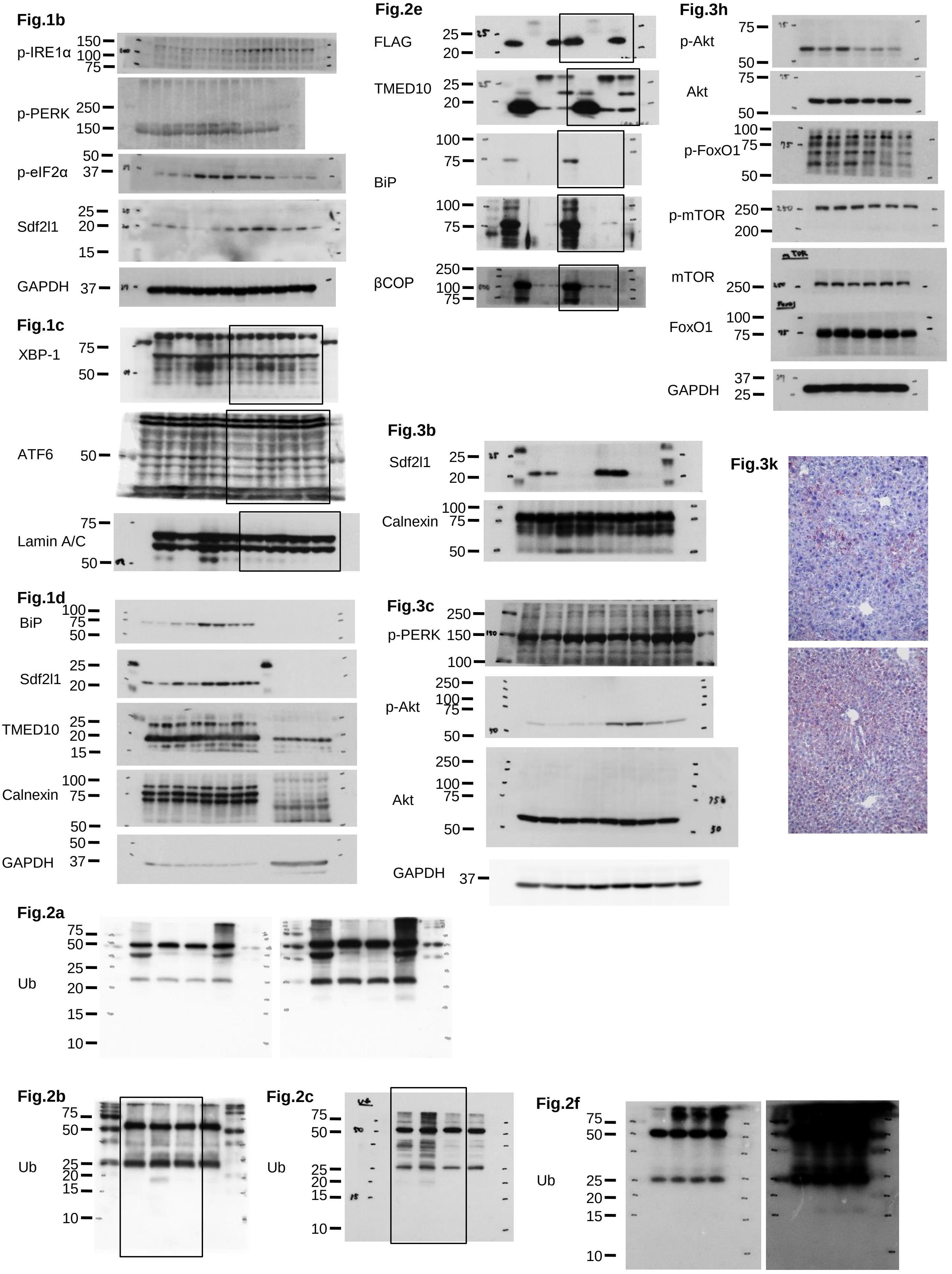

Supplementary Figure 11, continued

Fig.4a

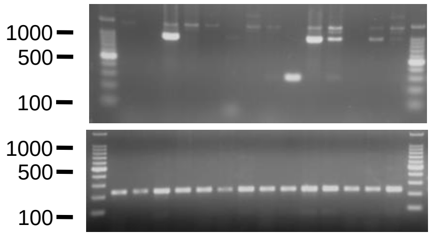

Fig.4i

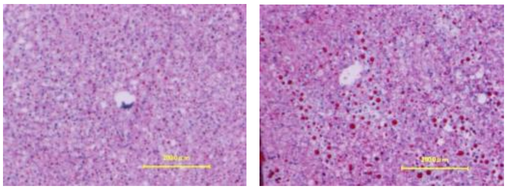

Fig.5a

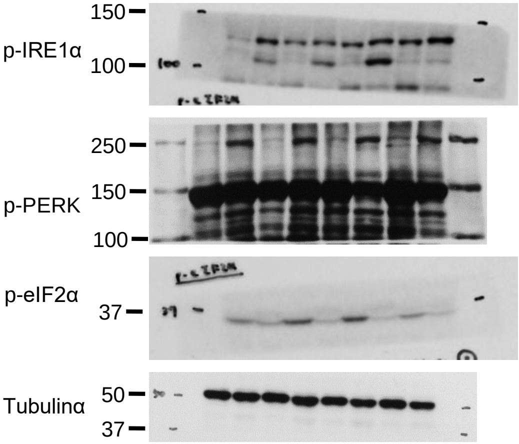

Fig.5c

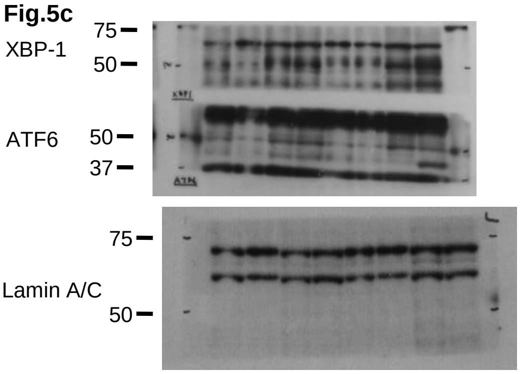

Fig.5e

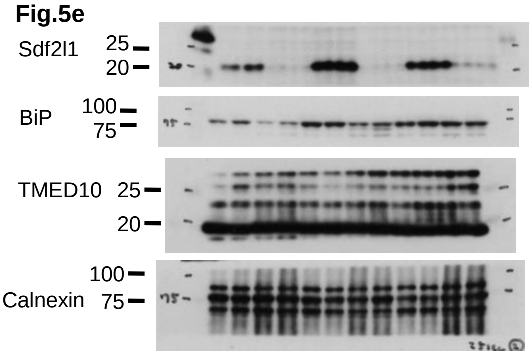

Fig.6b

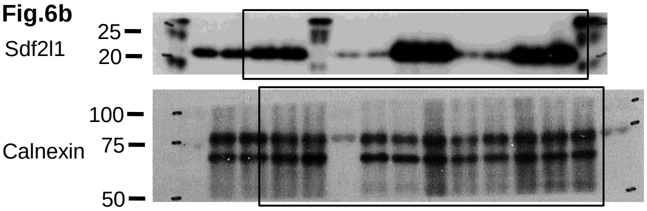

Fig.6c

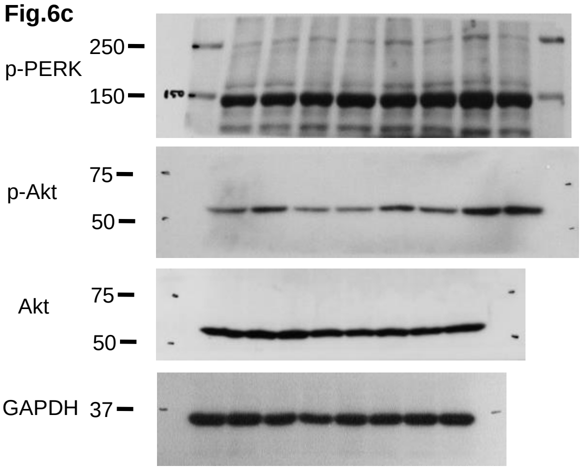

Fig.6h

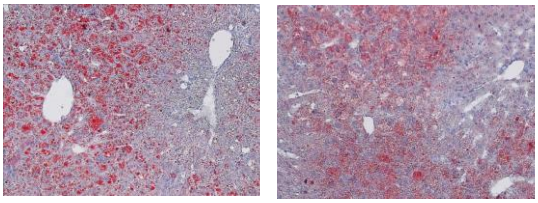

Supplementary Figure 11, continued

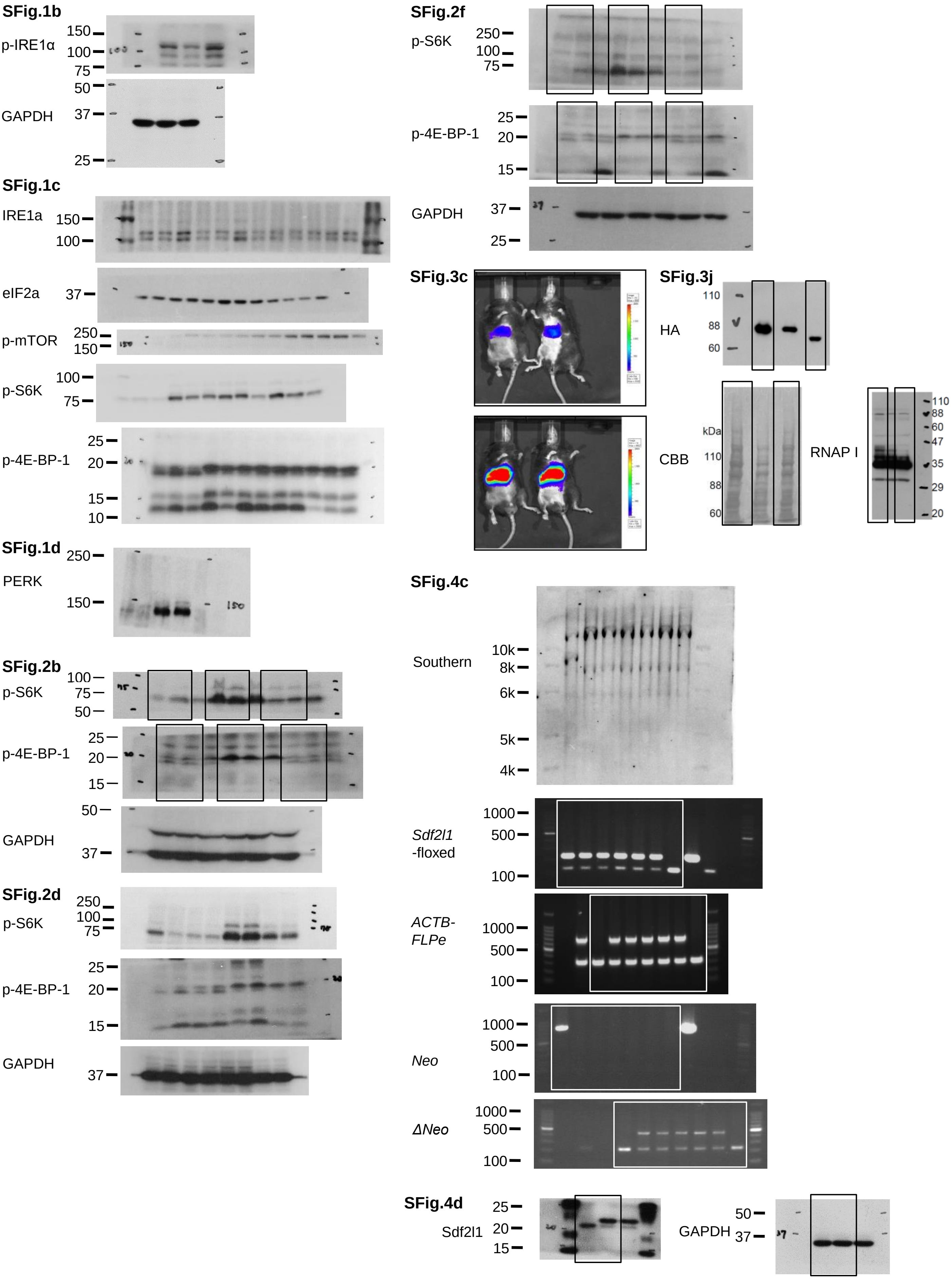

Supplementary Figure 11, continued

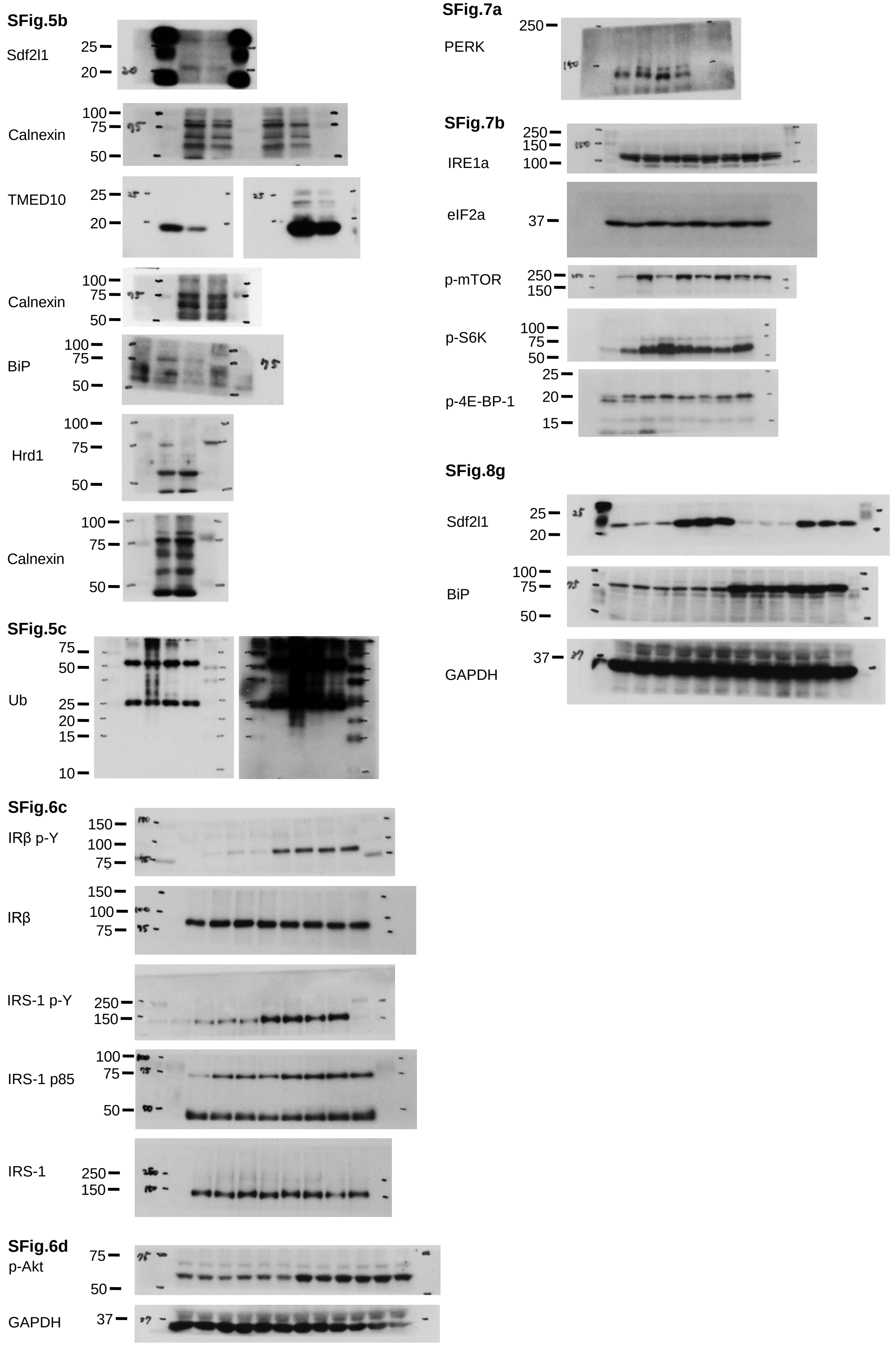

Supplementary Table 1

Genes up-regulated during feeding.

Fold change	Spot ID	Gene ID	Gene Symbol
11.31	A_55_P1997003	321018	*Serpina4-ps1*
10.99	A_55_P2004606	321018	*Serpina4-ps1*
8.35	A_51_P122246	76737	*Creld2*
8.30	A_51_P122246	76737	*Creld2*
8.28	A_55_P2107182	624219	*Gm6484*
8.27	A_51_P453043	78894	*Aacs*
8.24	A_55_P2111980	15496	*Hsd3b5*
8.17	A_51_P122246	76737	*Creld2*
8.10	A_51_P122246	76737	*Creld2*
7.99	A_51_P122246	76737	*Creld2*
7.93	A_51_P134142	226105	*Cyp2c70*
7.90	A_51_P122246	76737	*Creld2*
7.74	A_51_P122246	76737	*Creld2*
7.71	A_51_P122246	76737	*Creld2*
7.66	A_51_P122246	76737	*Creld2*
7.52	A_51_P122246	76737	*Creld2*
7.47	A_51_P491667	70377	*Derl3*
7.44	A_51_P189361	71839	*Osgin1*
6.07	A_55_P2121956	103988	*Gck*
5.97	A_51_P280446	64136	*Sdf2l1*
5.78	A_55_P2018666	21835	*Thrsp*
5.62	A_55_P1953103	67528	*Nudt7*
5.04	A_52_P306357	246691	*Prok1*
5.04	A_51_P418168	74840	*Manf*
4.66	A_55_P1975185	20775	*Sqle*
4.65	A_52_P136914	67528	*Nudt7*
4.55	A_55_P2150976	622384	*Fabp5l2*
4.48	A_51_P355943	192156	*Mvd*
4.43	A_55_P1966804	110196	*Fdps*
4.32	A_55_P2168267	--	--
4.28	A_55_P2041723	68041	*Mid1ip1*
4.25	A_55_P2122841	--	--
4.12	A_55_P1953387	16592	*Fabp5*
4.06	A_55_P2036547	15493	*Hsd3b2*
4.00	A_55_P2026761	--	--
3.91	A_52_P382149	13082	*Cyp26a1*

3.85	A_51_P397673	100102	*Pcsk9*
3.82	A_55_P2092219	71907	*Serpina9*
3.77	A_52_P2710	69049	*Cml5*
3.74	A_55_P2046411	--	--
3.70	A_55_P1986341	241041	*Gm4956*
3.38	A_52_P653825	64697	*Keg1*
3.32	A_51_P296487	16987	*Lss*
3.30	A_55_P2046408	--	--
3.30	A_66_P120125	27528	*D0H4S114*
3.26	A_52_P72237	11465	*Actg1*
3.23	A_55_P2114995	--	--
3.22	A_55_P2111855	74246	*Gale*
3.22	A_51_P209372	66234	*Sc4mol*
3.21	A_51_P395856	108114	*Slc22a7*
3.18	A_55_P2140751	--	--
3.18	A_55_P2028961	319554	*Idi1*
3.16	A_51_P386810	331026	*Gmppb*
3.16	A_51_P429366	55927	*Hes6*
3.14	A_55_P2076772	14828	*Hspa5*
3.14	A_55_P2025829	--	--
3.04	A_55_P2034227	--	--
3.02	A_66_P130647	100048479	*LOC100048479*
2.97	A_55_P2114994	--	--
2.93	A_55_P1961466	13190	*Dct*
2.90	A_55_P2158866	54369	*Nme6*
2.89	A_51_P431329	12350	*Car3*
2.88	A_51_P193336	53322	*Nucb2*
2.87	A_55_P2139087	434674	*Gm5631*
2.84	A_52_P164161	13121	*Cyp51*
2.84	A_51_P461429	13123	*Cyp7b1*
2.84	A_55_P2138306	--	--
2.83	A_55_P2114993	11465	*Actg1*
2.77	A_55_P2032966	208715	*Hmgcs1*
2.74	A_55_P2175065	100048053	*LOC100048053*
2.74	A_55_P2015734	74126	*Syvn1*
2.73	A_55_P1970597	11465	*Actg1*
2.72	A_55_P2142251	97114	*Hist2h3c2*
2.71	A_51_P380178	15903	*Id3*
2.71	A_55_P1970596	--	--

2.70	A_51_P300709	20810	*Srm*
2.70	A_52_P137371	15357	*Hmgcr*
2.69	A_52_P100252	14104	*Fasn*
2.68	A_55_P2108837	22146	*Tuba1c*
2.68	A_55_P1985984	11465	*Actg1*
2.68	A_51_P284244	66111	*Tmed3*
2.65	A_52_P624149	22027	*Hsp90b1*
2.64	A_55_P2228122	234757	*BC024137*
2.64	A_55_P2139089	434674	*Gm5631*
2.61	A_55_P2064547	22146	*Tuba1c*
2.61	A_52_P424462	67475	*Ero1lb*
2.61	A_51_P219505	338365	*Slc41a2*
2.59	A_55_P2318584	11833	*Aqp8*
2.59	A_51_P239750	16323	*Inhba*
2.59	A_51_P361620	--	--
2.58	A_52_P320193	94071	*Clec2h*
2.57	A_52_P164136	105171	*Arrdc3*
2.56	A_51_P188073	67838	*Dnajb11*
2.56	A_55_P1962736	27054	*Sec23b*
2.54	A_55_P2027102	50780	*Rgs3*
2.53	A_55_P2124712	234671	*Ces2*
2.53	A_51_P209319	110172	*Slc35b1*
2.51	A_51_P290207	231070	*Insig1*
2.50	A_55_P2120919	100043874	*Gm4703*
2.48	A_55_P2058864	17855	*Mvk*
2.47	A_55_P2003053	13190	*Dct*
2.42	A_55_P2002376	20810	*Srm*
2.42	A_52_P533146	13198	*Ddit3*
2.42	A_51_P297968	71853	*Pdia6*
2.42	A_52_P10041	11677	*Akr1b3*
2.42	A_52_P641282	71830	*Pdilt*
2.41	A_51_P328300	12304	*Pdia4*
2.41	A_55_P1977653	72333	*Palld*
2.40	A_52_P240542	15902	*Id2*
2.39	A_51_P296608	13197	*Gadd45a*
2.38	A_66_P137462	18194	*Nsdhl*
2.37	A_55_P2101696	14686	*Gnat2*
2.37	A_55_P2023114	15493	*Hsd3b2*
2.36	A_55_P2170881	71302	*Arhgap26*

2.35	A_55_P2045658	54369	*Nme6*
2.35	A_51_P196127	23971	*Papss1*
2.34	A_52_P318361	234671	*Ces2*
2.32	A_52_P228236	22042	*Tfrc*
2.32	A_52_P592951	66357	*Ostc*
2.31	A_66_P121446	666185	*Gm7969*
2.31	A_55_P2116496	22143	*Tuba1b*
2.31	A_52_P228236	22042	*Tfrc*
2.30	A_52_P228236	22042	*Tfrc*
2.29	A_52_P228236	22042	*Tfrc*
2.29	A_52_P228236	22042	*Tfrc*
2.29	A_52_P228236	22042	*Tfrc*
2.29	A_52_P228236	22042	*Tfrc*
2.28	A_52_P228236	22042	*Tfrc*
2.28	A_51_P463440	170439	*Elovl6*
2.27	A_55_P2169124	--	--
2.27	A_55_P2023637	96875	*Prg4*
2.27	A_51_P502614	67603	*Dusp6*
2.26	A_55_P2003513	15505	*Hsph1*
2.24	A_51_P272553	20893	*Bhlhe40*
2.24	A_51_P449995	12274	*C6*
2.23	A_55_P1972490	--	--
2.22	A_52_P228236	22042	*Tfrc*
2.21	A_55_P1998601	228993	*Slc17a9*
2.21	A_55_P2033250	14137	*Fdft1*
2.21	A_52_P627269	234669	*BC015286*
2.20	A_55_P2159264	16880	*Lifr*
2.19	A_55_P2043767	639477	*LOC639477*
2.19	A_52_P228236	22042	*Tfrc*
2.18	A_55_P2160686	21807	*Tsc22d1*
2.18	A_55_P2176963	15505	*Hsph1*
2.17	A_55_P2037787	100042341	*Gm3798*
2.16	A_55_P2066116	12051	*Bcl3*
2.16	A_51_P305843	66917	*Chordc1*
2.15	A_55_P2053491	71853	*Pdia6*
2.15	A_51_P189442	26876	*Adh4*
2.13	A_51_P341918	21807	*Tsc22d1*
2.12	A_55_P2098697	21928	*Tnfaip2*
2.12	A_55_P2109752	94181	*Nans*

2.12	A_55_P2078955	11833	*Aqp8*
2.11	A_55_P2156625	--	--
2.10	A_55_P2006677	666185	*Gm7969*
2.10	A_51_P282760	18627	*Per2*
2.10	A_55_P1961320	21753	*Tes*
2.09	A_55_P2138104	--	--
2.09	A_65_P20167	12282	*Hyou1*
2.08	A_55_P1973213	328162	*Trmt61a*
2.08	A_51_P452768	64385	*Cyp4f14*
2.08	A_55_P2102060	14864	*Gstm3*
2.07	A_55_P2387665	102123	*9130221J18Rik*
2.07	A_51_P216593	22393	*Wfs1*
2.07	A_51_P416419	12317	*Calr*
2.07	A_51_P403693	66663	*Uba5*
2.07	A_55_P2008907	11465	*Actg1*
2.06	A_55_P1968664	--	--
2.06	A_52_P106259	13649	*Egfr*
2.05	A_55_P2159934	66825	*Rnf186*
2.05	A_66_P129730	14827	*Pdia3*
2.05	A_55_P2072908	--	--
2.05	A_51_P431785	17930	*Myom2*
2.05	A_51_P216075	13200	*Ddost*
2.04	A_51_P299805	71706	*Slc46a3*
2.04	A_55_P2174953	11677	*Akr1b3*
2.04	A_55_P2153291	--	--
2.04	A_52_P106259	13649	*Egfr*
2.04	A_51_P111259	52004	*Cdk2ap2*
2.04	A_55_P2153292	227613	*Tubb2c*
2.04	A_52_P106259	13649	*Egfr*
2.04	A_52_P106259	13649	*Egfr*
2.03	A_55_P2008244	--	--
2.03	A_52_P384574	170459	*Stard4*
2.03	A_51_P185175	14228	*Fkbp4*
2.02	A_51_P277088	16005	*Igfals*
2.02	A_66_P134265	384198	*Gm1381*
2.01	A_55_P1959703	100042651	*Tubb2c-ps1*
2.01	A_55_P2055537	104681	*Slc16a6*
2.01	A_55_P1994032	22433	*Xbp1*
2.00	A_55_P2465382	--	--

2.00	A_55_P2083894	100042151	*Gm3697*

Results of microarray analysis (GEO accession: GSE59885), comparing global gene expression between a 24-hour fasted state and a 6-hour refed state in the liver of the control mice (*Ir* -floxed or *Ir* /*Foxo1* -floxed on a mixed background of C57BL/6 and FVB/N): transcripts up-regulated during refeeding, with expression ratio (refed/fasted) > 2.0 fold (n = 7).

Supplementary Table 2

Proteins detected by mass spectrometric analysis.

NSAF	Accession Number	Identified Proteins
0.002351	sp|P62274|RS29_MOUSE	Rps29
0.001338	sp|Q99KV1|DJB11_MOUSE	Dnajb11
0.001045	sp|P61082|UBC12_MOUSE	Ube2m
0.000968	sp|P61089|UBE2N_MOUSE	Ube2n
0.000914	sp|P47915|RL29_MOUSE	Rpl29
0.000866	sp|P84104|SRSF3_MOUSE	Srsf3
0.000823	sp|P23927|CRYAB_MOUSE	Cryab
0.000784	sp|O88456|CPNS1_MOUSE	Capns1
0.000645	sp|P61079|UB2D3_MOUSE	Ube2d3
0.000523	sp|P97315|CSRP1_MOUSE	Csrp1
0.000499	sp|Q791V5|MTCH2_MOUSE	Mtch2
0.000457	sp|Q9QY73|TMM59_MOUSE	Tmem59
0.000457	sp|P45376|ALDR_MOUSE	Akr1b1
0.000439	sp|Q9D1D4|TMEDA_MOUSE	Tmed10
0.000439	sp|Q9DBZ5|EIF3K_MOUSE	Eif3k
0.000392	sp|Q6PDM2|SRSF1_MOUSE	Srsf1
0.000378	sp|Q9D8M4|RL7L_MOUSE	Rpl7l1
0.000354	sp|Q9R0U0|SRS10_MOUSE	Srsf10
0.000343	sp|P97447|FHL1_MOUSE	Fhl1
0.000343	sp|Q9CPN8|IF2B3_MOUSE	Igf2bp3
0.000314	sp|Q8R4R6|NUP53_MOUSE	Nup35
0.000314	sp|O35682|MYADM_MOUSE	Myadm
0.000309	sp|Q8BMA6|SRP68_MOUSE	Srp68
0.000305	sp|P47740|AL3A2_MOUSE	Aldh3a2
0.000297	sp|O35382|EXOC4_MOUSE	Exoc4

0.000289	sp|Q9QYJ3|DNJB1_MOUSE	Dnajb1
0.000279	sp|Q8VH51|RBM39_MOUSE	Rbm39
0.00027	sp|P59326|YTHD1_MOUSE (+1)	Ythdf1
0.000268	sp|P32233|DRG1_MOUSE	Drg1
0.000264	sp|P97452|BOP1_MOUSE	Bop1
0.000261	sp|Q9D8V0|HM13_MOUSE	Hm13
0.000261	sp|Q9CYH6|RRS1_MOUSE	Rrs1
0.000261	sp|P18406|CYR61_MOUSE	Cyr61
0.000255	sp|Q60854|SPB6_MOUSE	Serpinb6
0.000255	sp|P35486|ODPA_MOUSE	Pdha1
0.000249	sp|P50580|PA2G4_MOUSE	Pa2g4
0.000246	sp|Q9EP69|SAC1_MOUSE	Sacm1l
0.000233	sp|Q9WV60|GSK3B_MOUSE	Gsk3b
0.000229	sp|P97346|NXN_MOUSE	Nxn
0.000229	sp|Q99LE6|ABCF2_MOUSE	Abcf2
0.000224	sp|Q9WVJ9|FBLN4_MOUSE	Efemp2
0.000224	sp|Q8BWY3|ERF1_MOUSE	Etf1
0.000224	sp|Q8VCM7|FIBG_MOUSE	Fgg
0.000222	sp|Q9WVR4|FXR2_MOUSE	Fxr2
0.000215	sp|O54784|DAPK3_MOUSE	Dapk3
0.000211	sp|P97855|G3BP1_MOUSE	G3bp1
0.000207	sp|Q3V3R1|C1TM_MOUSE	Mthfd1l
0.000207	sp|Q99LD4|CSN1_MOUSE	Gps1

Results of LC/MS/MS analysis, using a microsomal fraction of Sdf2l1-knockout MEF cells, expressed with Sdf2l1-FLAG and treated with tunicamycin, after immunoprecipitation with FLAG antibody or control mouse antibody: the peptides detected only in the sample immunoprecipitated with FLAG antibody, with NSAF > 0.0002.
