# Supplementary material for: Hepatic Sdf2l1 controls feeding-induced ER stress and regulates metabolism
Source: Nat Commun. 2019 Feb 27;10:947. doi: 10.1038/s41467-019-08591-6 (PMC6393527; doi:10.1038/s41467-019-08591-6)
Supplement: Supplementary file 2 — Reporting Summary [file 41467_2019_8591_MOESM2_ESM.pdf]

nature research

Corresponding author(s): Takashi Kadowaki and Kohjiro Ueki

Last updated by author(s): Dec 27, 2018

Reporting Summary

Nature Research wishes to improve the reproducibility of the work that we publish. This form provides structure for consistency and transparency in reporting. For further information on Nature Research policies, see Authors & Referees and the Editorial Policy Checklist.

Statistics

For all statistical analyses, confirm that the following items are present in the figure legend, table legend, main text, or Methods section.

n/a	Confirmed	
☐	☒	The exact sample size (*n*) for each experimental group/condition, given as a discrete number and unit of measurement
☐	☒	A statement on whether measurements were taken from distinct samples or whether the same sample was measured repeatedly
☐	☒	The statistical test(s) used AND whether they are one- or two-sided<br>*Only common tests should be described solely by name; describe more complex techniques in the Methods section.*
☒	☐	A description of all covariates tested
☐	☒	A description of any assumptions or corrections, such as tests of normality and adjustment for multiple comparisons
☐	☒	A full description of the statistical parameters including central tendency (e.g. means) or other basic estimates (e.g. regression coefficient) AND variation (e.g. standard deviation) or associated estimates of uncertainty (e.g. confidence intervals)
☐	☒	For null hypothesis testing, the test statistic (e.g. *F*, *t*, *r*) with confidence intervals, effect sizes, degrees of freedom and *P* value noted<br>*Give P values as exact values whenever suitable.*
☒	☐	For Bayesian analysis, information on the choice of priors and Markov chain Monte Carlo settings
☒	☐	For hierarchical and complex designs, identification of the appropriate level for tests and full reporting of outcomes
☒	☐	Estimates of effect sizes (e.g. Cohen's *d*, Pearson's *r*), indicating how they were calculated

Our web collection on statistics for biologists contains articles on many of the points above.

Software and code

Policy information about availability of computer code

Data collection	Not applicable.
Data analysis	EZ-R (ref. 66), as was described in the "Statistics" section in the Methods.

For manuscripts utilizing custom algorithms or software that are central to the research but not yet described in published literature, software must be made available to editors/reviewers. We strongly encourage code deposition in a community repository (e.g. GitHub). See the Nature Research guidelines for submitting code & software for further information.

Data

Policy information about availability of data

All manuscripts must include a data availability statement. This statement should provide the following information, where applicable:

- Accession codes, unique identifiers, or web links for publicly available datasets
- A list of figures that have associated raw data
- A description of any restrictions on data availability

All data supporting the findings of this study are available within the manuscript and its Supplementary Information files or are available from the authors upon reasonable request.

Field-specific reporting

Please select the one below that is the best fit for your research. If you are not sure, read the appropriate sections before making your selection.

☒ Life sciences ☐ Behavioural & social sciences ☐ Ecological, evolutionary & environmental sciences

Life sciences study design

All studies must disclose on these points even when the disclosure is negative.

Sample size	No statistical method was used to pre-determine sample size, in experiments using cultured cells and mice, as well as analysis of human samples.
Data exclusions	Data from mice getting ill after interventions (e.g. administration of an adenovirus) were excluded, otherwise all data were included. In the analysis of human samples, all data from eligible patients were included.
Replication	All the experiments using cultured cells and mice were supported by at least one additional experiment, whereas analysis using human samples was performed in a single cohort of patients.
Randomization	Body weight and blood glucose levels were matched among groups in experiments allocating mice to different interventions.
Blinding	The investigators were blinded to the genotype of mice or intervention to which mice were subjected when assessing metabolic phenotypes.

Reporting for specific materials, systems and methods

We require information from authors about some types of materials, experimental systems and methods used in many studies. Here, indicate whether each material, system or method listed is relevant to your study. If you are not sure if a list item applies to your research, read the appropriate section before selecting a response.

Materials & experimental systems

n/a	Involved in the study	
☐	☒	Antibodies
☐	☒	Eukaryotic cell lines
☒	☐	Palaeontology
☐	☒	Animals and other organisms
☐	☒	Human research participants
☒	☐	Clinical data

Methods

n/a	Involved in the study	
☒	☐	ChIP-seq
☒	☐	Flow cytometry
☒	☐	MRI-based neuroimaging

Antibodies

Antibodies used	Anti-Sdf2l1 antibody was generated by the authors, and the others were purchased from standard commercial sources or gifts from other researchers. As for those purchased, catalog numbers are provided in the "Antibodies" section in the Methods.
Validation	Anti-Sdf2l1 antibody was validated using samples from cultured cells and mouse tissues with Sdf2l1 knocked out or knocked down. The others were validated in reference to manufacturer's website or relevant citations.

Eukaryotic cell lines

Policy information about cell lines

Cell line source(s)	HEK293 cells, Fao cells, and NIH/3T3 cells were purchased from ECACC.
Authentication	The cell lines were purchased from the authorized source, and none of them were authenticated thereafter.
Mycoplasma contamination	None of the cell lines showed signs of mycoplasma contamination, and were not tested for it.
Commonly misidentified lines (See ICLAC register)	Not applicable.

Animals and other organisms

Policy information about studies involving animals; ARRIVE guidelines recommended for reporting animal research

Laboratory animals	8- to 10-week-old male mice were subjected to experiments, and they were housed under a 12-hour light/12-hour dark cycle and had free access to sterile water and food.
Wild animals	Not applicable.

Field-collected samples	Not applicable.
Ethics oversight	The Animal Care Committee of Graduate School of Medicine, the University of Tokyo.

Note that full information on the approval of the study protocol must also be provided in the manuscript.

Human research participants

Policy information about studies involving human research participants

Population characteristics	Patients with clinically suspected NAFLD who consented to liver biopsy.
Recruitment	Patients were prospectively recruited at the University of Tokyo Hospital from November 2011 to March 2014.
Ethics oversight	The University of Tokyo Medical Research Center Ethics Committee

Note that full information on the approval of the study protocol must also be provided in the manuscript.
